# Recommendations for the pharmacological treatment of treatment-resistant depression: A systematic review protocol

**DOI:** 10.1371/journal.pone.0267323

**Published:** 2022-04-19

**Authors:** Franciele Cordeiro Gabriel, Airton Tetelbom Stein, Daniela Oliveira de Melo, Géssica Caroline Henrique Fontes-Mota, Itamires Benício dos Santos, Camila da Silva Rodrigues, Mônica Cristiane Rodrigues, Renério Fráguas, Ivan D. Florez, Diogo Telles Correia, Eliane Ribeiro

**Affiliations:** 1 Departamento de Farmácia, Faculdade de Ciências Farmacêuticas, Universidade de São Paulo, São Paulo, São Paulo, Brasil; 2 Departamento de Saúde Coletiva, Universidade Federal de Ciências da Saúde de Porto Alegre, Porto Alegre, Rio Grande do Sul, Brasil; 3 Curso de Pós-graduação em Avaliação de Tecnologia em Saúde, Hospital Conceição, Porto Alegre, Rio Grande do Sul, Brasil; 4 Departamento de Ciências Farmacêuticas, Instituto de Ciências Ambientais, Químicas e Farmacêuticas, Universidade Federal de São Paulo, Diadema, São Paulo, Brasil; 5 Faculdade de Saúde Pública, Universidade de São Paulo, São Paulo, São Paulo, Brasil; 6 Laboratório de Neuro-imagem em Psiquiatria—LIM-21, Departamento e Instituto de Psiquiatria, Hospital das Clínicas, Faculdade de Medicina, Universidade de São Paulo, Divisão de Psiquiatria e Psicologia, Hospital Universitário, Universidade de São Paulo, São Paulo, São Paulo, Brasil; 7 School of Rehabilitation Sciences, McMaster University, Hamilton, Ontario, Canada; 8 Department of Pediatrics, University of Antioquia, Medellín, Colombia; 9 Pediatric Intensive Care Unit, Clinica Las Americas-AUNA, Medellin, Colombia; 10 Departamento de Psiquiatria e Psicologia da Faculdade de Medicina da Universidade de Lisboa, Lisboa, Portugal; International Hellenic University: Diethnes Panepistemio tes Ellados, GREECE

## Abstract

**Introduction:**

Depression is a serious and widespread mental health disorder. Although effective treatment does exist, a significant proportion of patients with depression fail to respond to antidepressant treatment trials, a condition named treatment-resistant depression. Efficient approach should be given this condition in order to revert the burden caused by depression. Clinical practice guidelines (CPGs) are evidence-based health promotion instruments to improve diagnosis and treatment. CPGs recommendations for treatment-resistant depression must be trustworthy. The objective of the proposed study is to systematically identify, appraise the quality of CPGs for the treatment of depression and elaborate a synthesis of recommendations for treatment-resistant depression of CPGs considered to be of high quality and with high quality recommendations.

**Methods and analysis:**

We will search the databases of organizations, such as PubMed, Embase, Cochrane Library, PsycInfo, and the Virtual Health Library, and organizations that develop CPGs. Three independent researchers will assess the quality of the CPGs and their recommendations using the AGREE II and AGREE-REX instruments, respectively. Given the identification of divergences and convergences as well as weak and strong points among high quality CPGs, our work may help developers, clinicians and eventually patients.

**Ethics and dissemination:**

No ethical approval is required for a systematic review, as no patient data will be used. The research results will be disseminated in conferences and submitted to a peer reviewed journal.

## Introduction

Depression is a serious medical illness that negatively affects behaviour. This condition causes feelings of sadness and/or loss of interest in everyday life activities. It is also a common condition, which affects more than 300 million people worldwide and is considered one of the most relevant public health problems in the 21st century [1]. Owing to its disabling nature, it can cause various professional, economic, social, and personal losses [2]. Patients with lower response to depression treatment have higher risk of severe outcomes including job loss, isolation, and suicide which may lead to an increased economic cost to society [3]. In addition, over the last years the number of depressed people has increased considerably, overloading health systems and generating a greater need for resource optimization [4].

There are several classes of antidepressants including serotonin reuptake inhibitors, serotonin and norepinephrine reuptake inhibitors, tricyclic antidepressants, noradrenergic and serotonergic antidepressants, monoamine oxidase inhibitors, and others, which are effective for medical treatment of depression [5]. Around only one third of patients will remit after a trial with a selective serotonin reuptake inhibitor (SSRI) and only 25% to 27% will remit after a subsequent trial with another antidepressant [6]. Consequently, a significant number of patients—up to 40%—will be treatment-resistant (i.e., failed to remit to at least two antidepressant trials) [6].

Treatment-resistant depression is difficult to manage, and results are usually poor, especially when unstandardized approaches are used [7–10]. In this respect, clinical practice guidelines (CPGs) are an essential tool for guiding clinical decisions. CPGs are supported by the best available evidence gathered through rigorous systematic reviews. In addition, CPGs consider additional key elements before providing a recommendation—such as costs, feasibility, patients’ values, and preferences—and aim to balance benefits and harms, among others key aspects [11, 12].

Several instruments are available to evaluate the methodological quality of CPGs [13, 14]. One of the most widely used is the Appraisal of Guidelines Research & Evaluation (AGREE II). This instrument provides a comprehensive, rapid, and robust assessment of CPGs [13, 15, 16]. The quality of CPGs for depression has been the focus of several reviews in recent years. For instance, in a recent review, only 4 out of 11 CPGs met the minimum quality benchmark after their assessment with the AGREE II tool [17]. Another review found that only 6 out of 27 CPGs could be classified as high-quality CPGs [18].

The low methodological quality of CPGs can cause inconsistencies among recommendations [19, 20]. In a study comparing the recommendations for pharmacotherapy and neurostimulation in the treatment of depression, a high degree of inconsistency was found in the recommendations for the second and third lines of treatment [21]. Discrepancies among recommendations for first-line treatments and for patients not responding to first-line treatment were also observed by MacQueen et al. [20], in a study analyzing CPGs used in primary healthcare for major depression, dysthymia, and minor depression.

High quality CPGs are crucial for developing recommendations that are based on the best evidence, with a transparent and trustworthy process that is implementable and acceptable by stakeholders and patients. However, it should be noted that high-quality CPGs, i.e., CPGs that obtain high scores on AGREE II assessment, do not necessarily guarantee credible and implementable recommendations [22]. In response to this gap, the AGREE Collaboration developed another instrument to evaluate the quality of the recommendations. The Appraisal of Guidelines Research and Evaluation-Recommendations Excellence (AGREE-REX) tool [23, 24], includes additional factors—such as clinical application, stakeholder values and preferences, and feasibility of implementation. These factors are considered by CPG developers for more feasible, credible, and implementable recommendations, and by guideline users interested in evaluating recommendation quality.

Although previous studies have evaluated the methodological quality of CPGs for the treatment of depression using AGREE II and the discrepancies among recommendations [18, 25–27], no study has evaluated the quality of their recommendations. However, doing so would be important for the development of CPGs by increasing the reliability of the recommendations, which could enhance evidence-based clinical practices implementation. Finally, there is a need to have reliable CPGs for treatment-resistant depression, as an effective treatment is a very common decision-making question among clinicians, and much inconsistency occurs in clinical practice, as the available CPGs do not have a strong recommendation. The scope of this proposal is to fulfill this lack in the management of depression as a public health problem. Mainly because these are patients who, with each therapeutic failure of pharmacological treatments, are more likely to have serious outcomes such as suicide.

Thus, the aim of this systematic review protocol is to identify and appraise the certainty of evidence of recommendations to the approach of treatment-resistant depression in CPGs.

## Materials and methods

### Literature search

A comprehensive bibliographic search will be conducted across the following databases: MEDLINE (through PubMed), Embase, Cochrane Library, PsycInfo, and the Virtual Health Library. The complete database search strategy is presented in S1 Appendix. A manual search will also be undertaken on the websites of organizations responsible for developing or compiling CPGs such as the Australian Clinical Practice Guidelines (clinicalguidelines.gov.au), the Brazilian Ministry of Health (saude.gov.br), the Canadian Agency for Drugs and Technologies in Health (cadth.ca), the Chilean Ministry of Health (librarinsal.cl/guides-clinicasauge/), the Colombian Ministry of Health and Social Protection (http://gpc.minsalud.gov.co/gpc/SitePages/default_gpc.aspx), the Guidelines International Network (https://guidelines.ebmportal.com/), the Institute for Clinical Systems Improvement (icsi.org), the GuíaSalud Portal (Spain) (guiasalud.es), the Scottish Intercollegiate Guidelines Network (sign.ac.uk), the National Institute for Health and Care Excellence (nice.org.uk), Guideline Central (https://www.guidelinecentral.com/library/), and the ECRI library (https://www.ecri.org/library/).

We will also refer to documents complementary to the identified CPGs, such as summaries of recommendations, documents intended for patient education, search strategies, and methodological manuals. The literature search will be limited to CPGs published in the last ten years due to new initiatives to understand and establish criteria to define and diagnose treatment-resistant depression.

### Eligibility criteria

The CPGs providing recommendations for the treatment- resistant depression in adults will be included in the study. We will consider all documents that provide recommendations for the pharmacological management of depression, regardless of the methodology used to develop the recommendations. We will exclude CPGs intended only for local use, (e.g., developed to be used in a hospital), or specifically for patients with comorbidities (e.g., CPGs for patients with cancer, dementia, or multiple sclerosis) due to the lack of certainty of evidence and inability to apply in these different contexts. Moreover, CPGs focused on nonpharmacological interventions for depression will also be excluded (e.g., psychotherapy and electroconvulsive therapy). We will include CPGs published in the last ten years. In case of two or more versions of the same CPG in this period, we will include only the updated version.

#### Protocol registration

The results of this study will be published in the form of a scientific paper that will be prepared according to the PRISMA 2020 guidelines [28].

### Selection of CPGs

**Fig 1:**
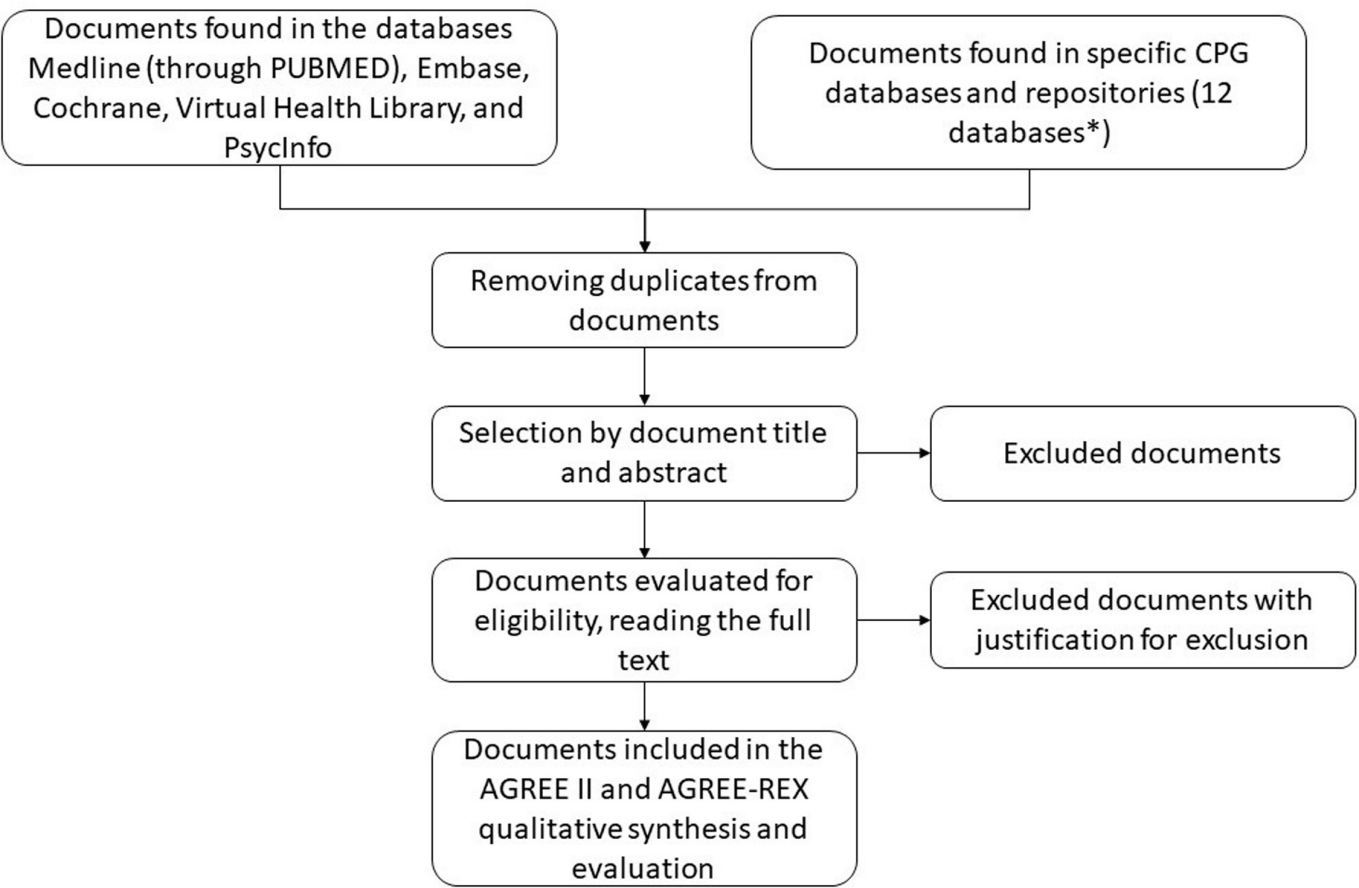


Fig 1 illustrates the planned CPG selection process. Duplicates will be removed using EndNote and the final reference list will be imported to the online review platform Rayyan® [29] for the screening and selection process.

The selection of CPGs will be conducted in two steps. First, titles and abstracts will be independently screened by two reviewers to verify their eligibility. Then, the two reviewers will also independently appraise the full text of potentially eligible documents. Discrepancies between reviewers in those two steps will be discussed and resolved by consensus, or by a third researcher if consensus is not achieved.

### Data extraction

Data extraction from the selected CPGs will be performed independently by two researchers using a piloted standard form on Google Forms®. The available data will be downloaded as an Excel spreadsheet and checked for agreement. Discrepancies will be resolved by consensus among the researchers and by a third researcher if consensus is not achieved.

The following data will be extracted from each included CPG: year of publication or updated version of the CPGs, classification of evidence, and institution or organization.

### Evaluation of the quality of the CPGs

The quality of the CPGs will be evaluated using the AGREE II instrument. The Portuguese version of the tool will be used [30]. It comprises 23 items organized into six domains: 1) scope and purpose, 2) stakeholder involvement, 3) rigour of development, 4) clarity of presentation, 5) applicability, and 6) editorial independence [14]. Each AGREE II item is scored using a 7-point Likert scale, where 1 indicates that there is no information for the AGREE II item and 7 indicates that the information is of the highest possible quality. Three reviewers (FCG, GCHFM, and IBS) will perform the quality assessment. More information regarding reviewer training is provided in the training subsection below.

An experienced investigator will train the reviewers using the AGREE II tool. Discrepancies between AGREE II items (2 points or more between reviewers) will be discussed among reviewers until a consensus is reached. If no consensus is reached, the disagreement will be resolved by a fourth senior researcher. Final scores per domain will be calculated by summing the scores from the three researchers per individual item in a domain and by scaling the total as a percentage of the maximum possible score for that domain. Therefore, each domain will be scored in a range from 0% to 100% [30].

There is no established threshold to consider a CPG of high quality according to AGREE II [24]. However, we considered that CPGs with a score higher than 60% in domain 3 (rigour of development) of AGREE II will be classified as high-quality, since this cut-off point has already been adopted by several authors [27, 31–33].

The quality assessment scores will be automatically calculated for each domain using the AGREE Plus Platform® [34]. According to the Agree II users’ Manual [30], the scores for each domain will be regarded as independent. Moreover, since some authors have highlighted how variable the overall scores in AGREE II can be and the manual recommends using the domains independently, we will not use the final overall domain for the analyses [25, 35].

### Evaluation of the quality of recommendations

The AGREE-REX tool will be used to assess the quality of the CPG recommendations [24]. We will apply the tool to all the pharmacological recommendations provided by the CPGs. AGREE-REX consists of nine items grouped into three domains: clinical applicability, values and preferences, and feasibility of implementation. Each item will be scored independently by three reviewers, using a 7-point Likert scale. When a discrepancy of 2 points or more occurs among evaluators, we will try to resolve it through discussion and consensus. If no consensus is reached, a fourth reviewer will decide the final score. Each appraiser’s notes will be inserted into a Google form®, and the results will be saved in a Microsoft Excel spreadsheet. Similar to the approach described for AGREE II, the final scores per domain will be obtained with values between 0 and 100% [34]. Recommendations with scores higher than 60% in domain 1 (clinical applicability) of the AGREE-REX will be considered as of high-quality using the same criteria considered for the quality of the CPGs. Each AGREE-REX question will receive scores from the three appraisers, final score will be defined by consensus in case of discrepancies, and a fourth, senior researcher will make a decision.

### AGREE II and AGREE-REX training

We will develop a training process based on the approaches described above [36–38]. The training will include a detailed discussion of the AGREE II and AGREE-REX manuals with instructions for their use and key articles that have used those methodologies [23]. The researchers will later make the training available on the AGREE Research Trust (2017) website [39]. As part of the training, the evaluators will assess the quality of CPGs for the treatment of chronic pain [40], Gaucher’s disease [41], and obesity [42]. We used the evaluation of these CPGs to calibrate the AGREE instruments. After the assessment, reviewers and trainers will discuss the scores and the discrepancies found. The trainers will provide feedback and answer questions posed by the reviewers. In the second stage, the team will evaluate two of the latest CPGs for hyperthyroidism and urinary tract infection [43, 44]. Later, a second discussion about the tool and discrepancies will be held [27].

### Strategy for data synthesis

Only CPGs considered to have high-quality (AGREE II domain 3 score ≥ 60%) with recommendations also categorized as high-quality (AGREE-REX domain 1 score ≥ 60%) will be the included in the data synthesis.

Domain 3 from AGREE II (rigour of development) will be used to classify a CPG as “high-quality”, since this is the most important item regarding the reliability of its recommendations [45]. Domain 1 from AGREE-REX will be used specifically to classify recommendations as having high quality. For the extraction of recommendations from the selected CPGs only two independent researchers will be used. This task will include extracting the quality of the evidence and strength of the recommendations. We will group the recommendations in two main topics, “terminology for resistant depression” and “recommended management strategies”. The terminologies and sequences of the therapeutic strategies will be compared between the CPGs and the strategies and terminologies that the CPGs had in common will be synthesized in a table.

### Patient and public involvement

No patients were involved in the development of this protocol.

## Discussion

With the identification of divergences and convergences between the recommendations of CPGs, as well as the identification of flaw points of CPGs for the pharmacological treatment of treatment-resistant depression we will be able to reveal areas deserving improvements. Such improvements will help developers of CPGs to enhance their processes and create both superior quality CPGs and recommendations. In addition, this will also increase the likelihood that the CPGs will be implemented, which will benefit patients as well as healthcare professionals.

This protocol has several strengths. First, we will conduct a comprehensive literature search in 17 databases and CPG repositories. Second, appraisers will undergo rigorous training in the AGREE II and AGREE-REX tools. This will increase the reliability of the results. Third, assessments will be performed independently by three researchers, minimizing the subjectivity of the intrinsic evaluation method and thus, improving reliability. The strengths and limitations of each CPG will be thoroughly evaluated during the data collection and analysis process.

However, the study had some limitations as well. Treatment of depression requires a multidimensional approach, not only pharmacological treatment. We only plan to assess CPGs on pharmacological treatment, and therefore, will not be able to draw conclusions for CPGs covering exclusively other treatment alternatives (e.g., psychotherapy and electroconvulsive therapy). Furthermore, although we are planning to perform manual searches of many specific websites, we cannot guarantee that relevant CPGs are not missed.

Nevertheless, these possible limitations do not lessen the relevance of the proposed protocol, particularly considering the comprehensive literature search, rigorous training in the AGREE II and AGREE-REX tools, and assessment by multiple researchers in order to adequately appraise the certainty of evidence of CPGs addressing the management of treatment-resistant depression.

## Supporting information

S1 Appendix Complete database search strategy. (DOCX)
